# Role of STN1 and DNA Polymerase α in Telomere Stability and Genome-Wide Replication in Arabidopsis

**DOI:** 10.1371/journal.pgen.1004682

**Published:** 2014-10-09

**Authors:** Elisa Derboven, Heinz Ekker, Branislav Kusenda, Petra Bulankova, Karel Riha

**Affiliations:** 1 Gregor Mendel Institute, Austrian Academy of Sciences, Vienna, Austria; 2 Campus Science Support Facilities, Next Generation Sequencing Facility, Vienna, Austria; 3 Central European Institute of Technology, Masaryk University, Brno, Czech Republic; Texas A&M University, United States of America

## Abstract

The CST (Cdc13/CTC1-STN1-TEN1) complex was proposed to have evolved kingdom specific roles in telomere capping and replication. To shed light on its evolutionary conserved function, we examined the effect of STN1 dysfunction on telomere structure in plants. STN1 inactivation in *Arabidopsis* leads to a progressive loss of telomeric DNA and the onset of telomeric defects depends on the initial telomere size. While EXO1 aggravates defects associated with STN1 dysfunction, it does not contribute to the formation of long G-overhangs. Instead, these G-overhangs arise, at least partially, from telomerase-mediated telomere extension indicating a deficiency in C-strand fill-in synthesis. Analysis of hypomorphic DNA polymerase α mutants revealed that the impaired function of a general replication factor mimics the telomeric defects associated with CST dysfunction. Furthermore, we show that STN1-deficiency hinders re-replication of heterochromatic regions to a similar extent as polymerase α mutations. This comparative analysis of *stn1* and *pol α* mutants suggests that STN1 plays a genome-wide role in DNA replication and that chromosome-end deprotection in *stn1* mutants may represent a manifestation of aberrant replication through telomeres.

## Author Summary

Telomeres form an elaborate nucleoprotein structure that may represent an obstacle for replication machinery and renders this region prone to fork stalling. CST is an evolutionary conserved complex that was originally discovered to specifically act at telomeres. Interestingly, the function of CST seems to have diverged in the course of evolution; in yeast it is required for telomere protection, while in mammals it was proposed to facilitate replication through telomeres. In plants, inactivation of CST leads to telomere deprotection and genome instability. Here we show that the telomere deprotection in Arabidopsis deficient in STN1, one of the CST components, is consistent with defects in telomere replication and that STN1 phenotypes can be partially phenocopied by an impairment of a general replication factor, DNA polymerase α. In addition, we provide evidence that STN1 facilitates re-replication at non-telomeric loci. This suggests a more general role of CST in genome maintenance and further infers that its seemingly specific function(s) in telomere protection may rather represent unique requirements for efficient replication of telomeric DNA.

## Introduction

Telomeres form a specialized type of chromatin that protects the native chromosome ends from being perceived as DNA double strand breaks (DSB) and from eliciting a DNA damage response (DDR). Telomeres also play an important role in genome duplication; their *de novo* synthesis by telomerase counteracts the end-replication problem caused by the inability of the conventional DNA replication machinery to fully duplicate the ends of linear chromosomes. In the vast majority of eukaryotes, the telomeric DNA consists of TG/CA-rich repeats and terminates with a 3′ TG-rich protrusion, the so called G-overhang. Telomeric DNA serves as a binding platform for a set of evolutionary conserved proteins whose function is to support telomere protection and replication. However, studies across multiple model organisms have revealed, in some cases, remarkable differences concerning the utilization and necessity of these conserved factors in telomere-related processes [1], [2].

The protection of mammalian telomeres largely depends on the shelterin complex [3]. One of the functions of shelterin is to promote the formation and stabilization of t-loops, which are lariat structures produced by intrastrand invasion of the G-overhang into the duplex telomeric region [4], [5], [6]. T-loops are proposed to mediate chromosome capping by physically sequestering the G-overhang thereby limiting the access of DNA repair and processing factors to the chromosome ends. Although t-loops have been found in a variety of organisms, they do not seem to form in budding yeast. Instead, G-overhangs in *Saccharomyces cerevisiae* are protected by the CST complex, an RPA-like particle consisting of the subunits Cdc13, Stn1 and Ten1 [7]. CST specifically binds to the telomeric G-overhang via OB-folds, and deletion of any subunit is lethal due to DNA damage checkpoint activation and massive nucleolytic resection, which is mainly mediated by exonuclease 1 (Exo1) [8], [9], [10], [11]. In addition, CST is crucial for telomere replication by facilitating the recruitment of telomerase and DNA polymerase α to the chromosome termini, and thus coordinating G-overhang extension by telomerase with the fill-in synthesis of the complementary C-strand [12], [13], [14].

A complex analogous to yeast CST was recently found in vertebrates [15], [16]. It consists of the proteins STN1 and TEN1 that are orthologous to the yeast counterparts and of the CTC1 subunit, which shows little sequence homology to Cdc13, but seems to mediate similar functions. As in yeast, protein interaction and structural studies demonstrated a similarity between human CST and RPA [16], [17], [18]. Furthermore, human CST binds ssDNA with a preference for the telomeric G-strand sequence [16], [18]. However, unlike in yeast, the consequences of CST inactivation in mammals are not so detrimental. CTC1-null mice are viable, but have a greatly reduced lifespan due to bone marrow failure and G2/M checkpoint arrest in haematopoietic stem cells [19]. Down-regulation of CST components in human cell lines by siRNA led to a range of relatively mild telomere-related defects whose extent was dependent on the cell line and experimental conditions [15], [16], [18], [20], [21], [22]. Importantly, these studies revealed that CST facilitates replication through duplex telomeric region and functions in the C-strand fill-in reaction and G-overhang maturation [20], [21], [23], [24]. This led to the suggestion that mammalian CST is primarily involved in telomere replication, but not directly in protection. Thus, data from yeast and mammals imply that CST has evolved kingdom specific roles at telomeres [25].

The CST complex is also present in plants and all three subunits were identified and functionally characterized in *Arabidopsis thaliana* [15], [26], [27]. Null mutations in either CTC1, STN1 or TEN1 have an immediate impact on the telomere structure, resulting in shorter, heterogeneous telomeres, elongated G-overhangs, aberrant telomere recombination and chromosome end-to-end fusions. While mutant plants are viable, they exhibit retarded growth and reduced fertility. These defects are apparently caused by increased cell death of meristematic stem cells triggered by an ATR-mediated DNA damage response [28], [29]. Telomere dysfunction observed in *Arabidopsis* CST-deficient mutants led to the suggestion that the plant CST complex functions in both chromosome end protection and telomere replication, and may therefore represent an evolutionary bridge between budding yeast and mammals [15], [30]. This interpretation would further imply that telomere protection and replication represent ancient functions of CST and that the telomere-protective role was lost in animals. Therefore, understanding the mechanism by which CST contributes to telomere stability in plants is important to fully decipher the role of this complex in eukaryotic genome maintenance.

In this study we asked the question whether and how CST contributes to the protection of *Arabidopsis* telomeres by elucidating the processes that cause telomere dysfunction and genome instability in *stn1* mutants. The data presented here and published in our previous paper [31] suggest that the onset of telomere deprotection phenotypes in STN1-deficient plants is gradual, and correlates with the progressive loss of telomeric sequence that is only partially counteracted by telomerase. We show that the bulk of STN1-depleted telomeres are not exposed to an excessive resection by EXO1, although the nuclease promotes genome instability in *stn1* mutants, possibly by processing critically short telomeres generated through defects during telomere replication. Interestingly, malfunction of a general replication factor (DNA polymerase α) produces telomeric defects similar to STN1 dysfunction. Furthermore, we present evidence demonstrating a role of STN1 in replication of non-telomeric loci. This suggests that CST plays a broader role in DNA replication and that the seemingly specific function(s) of this complex in telomere protection may reflect the sensitivity of this genomic region to replication stress.

## Results

### STN1 deficiency results in a gradual onset of telomere dysfunction

**Figure 1:**
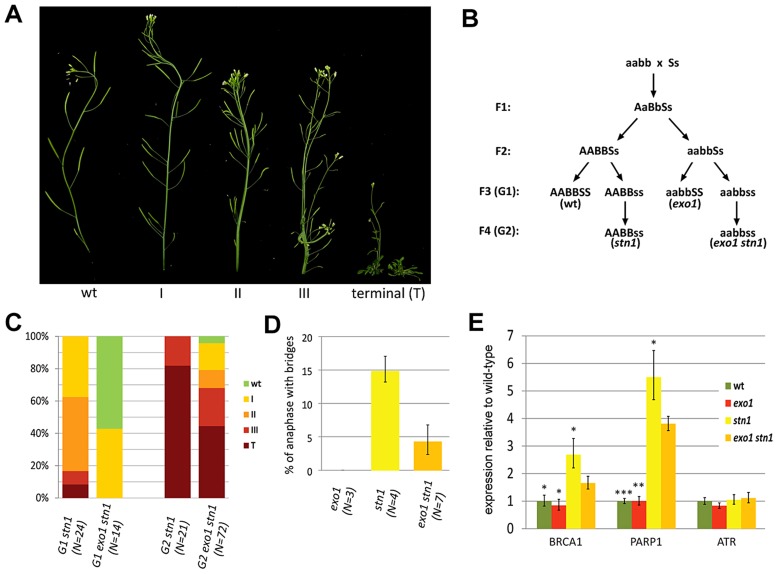
EXO1 aggravates the defects associated with STN1 depletion. (A) Phenotypic categories of *Arabidopsis stn1* mutants organized according to the severity of the observed growth defects. (B) Crossing scheme used to generate *exo1 stn1* mutants and control lines. Wild type and mutant alleles are indicated by capital and small letters, respectively (A – *EXO1A*, B – *EXO1B*, S – *STN1*). (C) Frequency of phenotypic categories in *stn1* and *exo1 stn1* mutant populations. The G2 populations were derived from the healthiest plants of the G1 generations. Number of plants scored is indicated in parentheses. (D) Frequency of anaphases with bridges in floral tissues. Number of plants scored is indicated in parentheses; at least 200 hundred anaphases were scored for each plant. The reduction of anaphase bridges in *stn1 exo1* compared to *stn1* mutants is highly significant (p<0.0001; two-tailed Student's t-test). (E) Expression of DDR genes in floral organs measured by qRT-PCR. Error bars represent SDs from three biological replicates; the asterisks denote a statistically significant difference to the *exo1 stn1* samples (Student's t-test; *p<0.05; **p<0.001; ***p<0.0001).

In our previous work we noticed that telomere dysfunction in *Arabidopsis stn1* mutants is less pronounced in plants with longer telomeres [31]. These data were obtained by analyzing *stn1* mutants derived from a cross between ecotypes with short (Col-0, 2–4 kb) and long (Ws, 4–8 kb) telomeres. The resulting first generation (G1) *stn1_(W/C)_* mutants displayed only mild growth defects and a low frequency of anaphase bridges. However, the severity of growth defects and genome instability increased in the subsequent generations [31] (Figure S1). This coincided with progressive telomere shortening. Although the *stn1_(C)_* mutants in a pure Col-0 background exhibit profound developmental defects already in G1, as in the case of *stn1_(W/C)_* plants, these defects worsened in G2 and G3 generations (Figures 1A and S1). To quantitatively describe the occurrence of these defects, we divided plants into five phenotypic categories from wt-looking plants (wt) to stunted plants unable to produce seeds (terminal). The intermediate phenotypes were characterized by altered flower phylotaxy (I), stem fasciation and more pronounced defects in phylotaxy (II), and massive stem fascination, aberrant leaf development and reduced fertility (III) (Figure 1A). Identical phenotypes were described in Arabidopsis *tert* mutants and are hallmarks of telomere dysfunction [32]. While the majority of G1 *stn1_(C)_* mutants exhibited phenotypes I and II, the bulk of G2 and G3 *stn1_(C)_* plants were terminal (Figure S1). The earlier onset of developmental phenotypes in *stn1_(C)_* plants inversely correlated with telomere length as G1 *stn1_(C)_* and *stn1_(W/C)_* telomeres were on average 2,2 and 2,8 kb, respectively. We also detected a further decline in the amount of telomeric DNA by dot blot hybridization in G2 and G3 *stn1_(C)_* (Figure S1D). These data indicate that the growth defects in *Arabidopsis stn1* mutants are caused by loss of telomeric DNA and that *STN1* disruption does not lead to immediate telomere dysfunction.

### STN1 depleted telomeres are processed by EXO1

One hallmark of telomere deprotection is the exposure of the chromosome termini to nuclease degradation. CST-deficient *Arabidopsis* exhibit very heterogeneous telomeres containing long G-overhangs, which may result from resection of unprotected telomeres by nuclease(s)[15], [26], [27]. *S. cerevisiae* telomeres depleted of Cdc13 undergo massive resection by Exo1 [11] and EXO1 orthologues degrade the telomeric C-strand and contribute to the telomere length heterogeneity in *Arabidopsis ku80* mutants [31]. To assess the effect of EXO1 on STN1-null telomeres, we analyzed *exo1a exo1b stn1* mutants where both *Arabidopsis* EXO1 paralogues have been disrupted (hereafter referred to as *exo1 stn1* mutants; Figure 1B). Inactivation of EXO1 largely rescued the gross developmental defects in G1 *exo1 stn1* and most plants in the mutant population were fully fertile. In contrast, G1 *stn1* plants derived from the same cross exhibited severe growth defects (Figure 1). This trend was also apparent in the second generation, where most G2 *stn1* mutants reached a terminal phenotype, while ∼1/3 of G2 *exo1 stn1* plants were still fertile and developed milder growth defects. In accordance with this result, we also observed a substantial decrease in anaphase bridges after EXO1 depletion (Figure 1D). It has been previously reported that CST dysfunction elicits a chronic DNA damage response that is characterized by transcriptional upregulation of a number of DNA repair genes including PARP1 and BRCA1 [28]. Expression of these genes is reduced in *exo1 stn1* plants, which further demonstrates that EXO1 exacerbates genome instability and DNA damage response after STN1 loss (Figure 1E).

**Figure 2:**
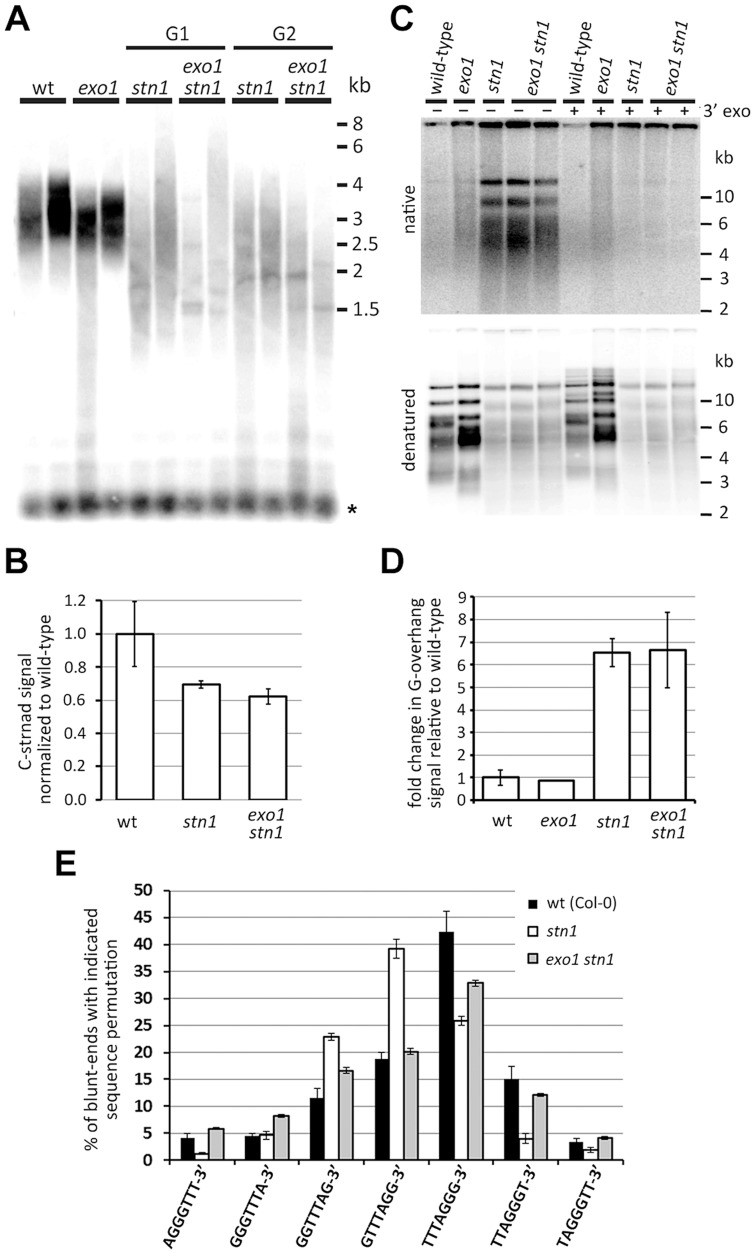
Effect of EXO1 on the structure of STN1-depleted telomeres. (A) TRF analysis; the asterisks indicate signal from interstitial telomeric DNA. (B) Quantification of the telomeric C-strand by dot-blot hybridization in G1 *stn1* and G1 *exo1 stn1* plants. Error bars show SDs from two independent samples; the P-value was calculated using a Student's t-test. (C) G-overhang analysis by the *in gel* hybridization technique. DNA samples pretreated with T4 DNA polymerase to remove 3′G-overhangs are indicated (3′ exo). The gels were first hybridized under nondenaturing conditions (top panels) and then denatured and hybridized again (bottom panels). (D) Quantification of G-overhang signals from a native gel. Error bars represent SDs from three (wt) and four (*stn1*; *stn1 exo1*) independent samples. (E) Frequency of telomeric sequence permutations forming the termini of blunt-ended telomeres. Error bars indicate SDs from five (wild-type) or four (*stn1*, *exo stn1*) biological replicates. Wild-type data are from [31].

We next wanted to know whether STN1-depleted telomeres are subject to EXO1-resection. Surprisingly, terminal restriction fragment (TRF) analysis and quantification of telomeric DNA by dot-blot hybridization did not reveal any discernable difference between *stn1* and *exo1 stn1* (Figure 2A,B). These data indicate that telomere shortening and length heterogeneity are not caused by EXO1 activity. To directly assay for telomere resection, we compared the relative G-overhang size in *stn1* and *exo1 stn1* plants by the *in gel* hybridization technique (Figure 2C,D). We observed a ∼6 fold increase in G-overhang signal in both mutants, showing that the excessive single-strandedness of telomeres in STN1-deficient plants is not due to EXO1. This data is supported by the observation that inactivation of EXO1 did not reduce t-circle excision in *stn1* mutants (Figure S2), a phenomenon caused by increased telomeric resection and recombination [31].

We have previously reported that only ∼50% of telomeres in *Arabidopsis* contain a long G-overhang, while the remaining telomeres are blunt- or nearly blunt-ended [31]. Furthermore, we showed that the blunt-ended telomeres preferentially terminate with the TTTAGGG-3′ permutation. Although the telomeric blunt-ends remain intact in *stn1* mutants, the preference of the terminal sequence shifts to GTTTAGG-3′. We proposed that the blunt-ended telomeres represent chromosome termini replicated by the leading strand machinery, whereas the G-overhang containing telomeres are the result of the lagging strand replication. Due to semiconservative DNA replication, the telomeric strand synthesized by the lagging strand-mechanism serves in the next round of replication as a template for the synthesis of a blunt-ended telomere [31]. Hence, the sequence preference of the telomeric blunt-ends likely reflects the processes involved in maturation of the lagging strand (G-overhang containing) telomeres. To examine whether the sequence alteration in STN1-depleted plants is caused by EXO1, we quantified the frequency of telomeric permutations forming the chromosome termini in *stn1* and *exo1 stn1* mutants by adaptor ligation-mediated PCR coupled with Illumina sequencing [31]. We confirmed our previous data showing that STN1 deficiency alters the frequency of terminal permutations (Figure 2E). Concomitant inactivation of EXO1 shifted the preference from GTTTAGG-3′ back to TTTAGGG-3′ and led to a permutation frequency profile similar to the one observed in wild-type plants (Figure 2E). This indicates that STN1-depleted telomeres, or at least a fraction of them, are subject to a limited degradation by EXO1. This processing does not grossly contribute to the single-strandedness of telomeres, but is detectable as a sequence alteration of the chromosome termini in *stn1* plants.

### Telomerase partially contributes to G-overhang extension in *stn1* mutants

**Figure 3:**
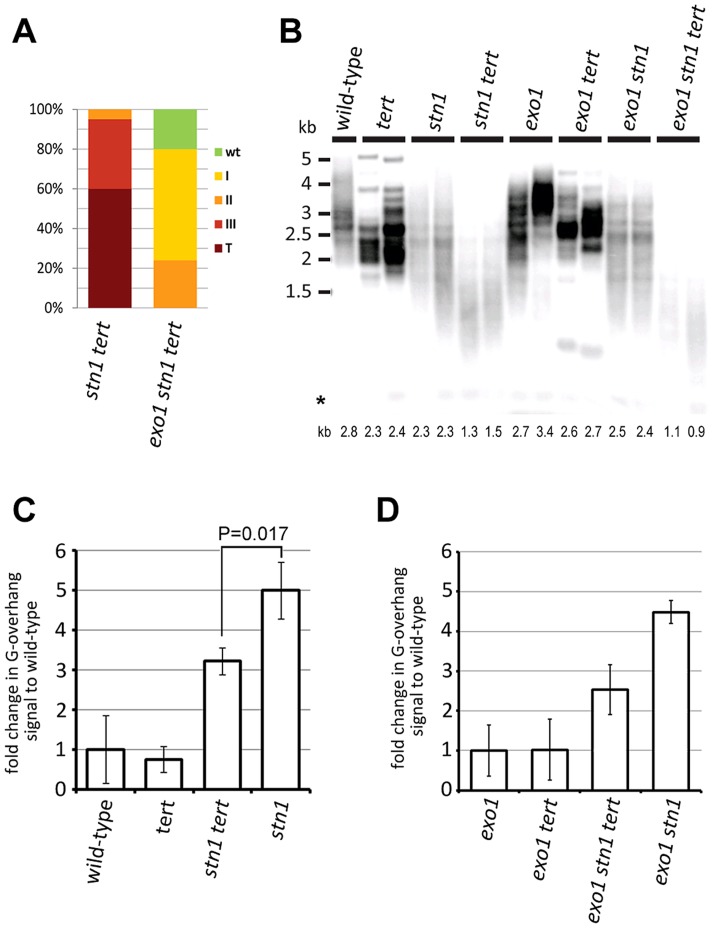
STN1 acts in the C-strand fill-in synthesis after G-strand extension by telomerase. (A) Frequency of phenotypic categories in G1 *stn1 tert* and G1 *exo1 stn1 tert* populations. (B) TRF analysis; the asterisks indicate signal from interstitial telomeric DNA. Average telomere length for each sample calculated by TeloTool is shown below the autoradiogram (C,D) Quantification of G-overhang signals from native gels (Figure S3). Errors represent SDs from three independent samples (two samples in the case of *exo1 stn1 tert*). Significance difference is indicated (two-tailed Student's test).

The extended G-overhangs in *stn1* plants may result from an inefficient fill-in synthesis of the telomeric C-strand after G-overhang elongation by telomerase. To test this hypothesis, we generated telomerase-null *stn1* and *exo1 stn1* mutants that carry a disruption of the telomerase reverse transcriptase (*TERT*) gene (Figure S3). Loss of telomerase exacerbated the growth defects seen in *stn1* mutants and the majority of the *stn1 tert* plants exhibited a terminal phenotype (compare charts in Figure 3A and Figure 1C). Inactivation of EXO1 significantly alleviated this growth retardation and *exo1 stn1 tert* plants developed much milder defects (Figures 3A and S3). This observation corroborates the analysis of *exo1 stn1* mutants and demonstrates that EXO1 activity is detrimental in the absence of STN1. Comparison of the telomere length among siblings outsegrgating from STN1^+/−^ TERT^+/−^ and *exo1 STN1^+/−^ TERT^+/−^* parents revealed that telomeres in *stn1 tert* and *exo1 stn1 tert* are much shorter than in the corresponding telomerase proficient controls (Figure 3B). These data argue that telomerase partially compensates for the loss of telomeric DNA in *stn1* mutants and mitigates the developmental defects ensuing from telomere dysfunction and genome instabilities.

We next tested whether telomerase activity contributes to the elongated G-overhangs in *stn1* mutants by performing an *in gel* hybridization assay. Quantification of the autoradiograms showed that telomerase-null plants have reduced G-overhangs in both *stn1* and *exo1 stn1* plants (Figures 3C,D and S3). This suggests that the G-strand extension by telomerase is partially uncoupled from synthesis of the complementary C-strand, implying that STN1 facilitates the C-strand fill-in synthesis.

### Impairment of DNA polymerase α leads to a subset of phenotypes identical to STN1 depletion

Inactivation of telomerase revealed that ∼1 kb of telomeric DNA is lost within one generation in *stn1 tert* mutants (compare *tert* and *stn1 tert* in Figure 3B), which is substantially more than the amount of telomeric DNA lost due to the end-replication problem in *tert* mutants (0.25 kb per generation) [33]. Interestingly, inactivation of EXO1 has no effect on the rate of shortening, indicating that mechanisms other than nuclease resection may contribute to this process. Recent studies in human cell lines suggested that CST associates with DNA polymerase α and facilitates the replication through human telomeres [20], [21], [23], [34], [35]. Hence, the telomere shortening in *Arabidopsis stn1* mutants may be due to inefficient replication through the telomeres, resulting in terminal deletions of telomeric DNA.

**Figure 4:**
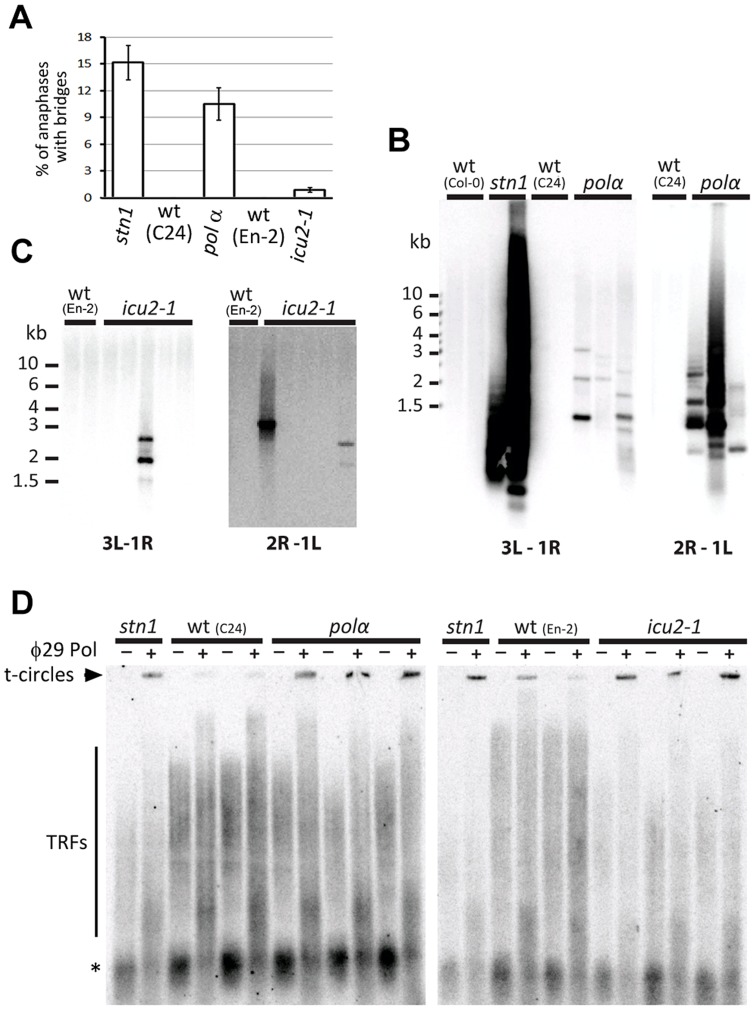
Impaired function of DNA polymerase α results in telomere deprotection. (A) Frequency of anaphases with bridges in floral tissues. (B,C) Amplification of chromosome end-to-end fusions by PCR in *pol α* and *icu2-1* mutants. Two combinations of subtelomeric primers specific for different chromosome arms (3L+1R and 2R+1L) were used. PCR products were detected by Southern hybridization with a telomeric probe. (D) Increased level of t-circles in *pol α*and *icu2-1* mutants measured by TCA. Signals from t-circles (arrowhead), TRFs, and interstitial telomeric DNA (asterisks) are indicated.

If STN1 functions with DNA polymerase α in telomere replication, impairment of DNA polymerase α may result in telomere-related phenotypes similar to the ones described in *Arabidopsis cst* mutants. To test this prediction, we analyzed plants that carry hypomorphic mutations in the catalytic subunit of DNA polymerase α. These alleles, named *pol α* and *icu2-1* were recovered in genetic screens for deregulated gene silencing and contain single amino acid substitutions in the evolutionarily conserved C-terminal domain (Figure S4A) [36], [37]. While both mutants are fertile, the stronger *pol α* allele causes severe growth retardation and developmental defects that are reminiscent of *stn1* mutants of categories II and III (Figure S4B). Plants carrying the *icu2-1* mutation develop only very mild abnormalities and are similar to *stn1* plants displaying the category I phenotype. Cytogenetic analysis revealed anaphase bridges in *pol α* as well as *icu2-1* mutants, indicating that both mutants suffer from ongoing genome instability (Figure 4A). The frequency of anaphase bridges was higher in *pol α* mutants, which correlates with the more severe developmental abnormalities.

Since polymerase α deficiency is expected to cause genome-wide chromosome instabilities, we next determined whether some of the anaphase bridges detected in *pol α* and *icu2-1* mutants arise from chromosome-end-to-end fusions by using a fusion PCR strategy that utilizes primers specific to subtelomeric regions [38]. We readily detected strong signals derived from telomeric fusions in *stn1* samples, and weaker, but reproducible signals in *pol α* plants. Cloning and sequencing the PCR products verified that these represent chromosome end-to-end fusions and contain telomeric DNA. The *icu2-1* samples also yielded specific products, albeit not in all analyzed samples (Figures 4B,C). This correlates with the lower frequency of anaphase bridges and milder phenotype of the *icu2-1* plants. Another hallmark of telomere dysfunction exhibited by *Arabidopsis stn1* mutants is aberrant telomeric recombination, manifested through the excision of extrachromosomal t-circles. To examine whether this is also the case in DNA polymerase α mutants, we analyzed t-circle abundance with the t-circle amplification assay [39]. We detected elevated levels of t-circles in both *pol α* and *icu2-1* plants when compared to their respective wild-type controls (Figure 4D).Together, the presence of chromosome end-to-end fusions and increased t-circle excision demonstrate that an impaired function of DNA polymerase α results in telomere-dysfunction similar to inactivation of STN1.

**Figure 5:**
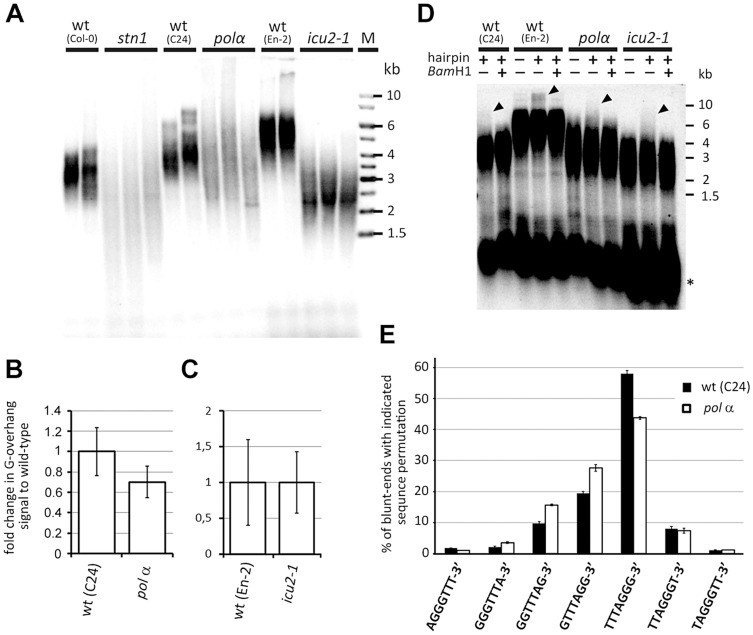
Telomere structure in DNA polymerase α mutants. (A) TRF analysis. (B,C) Quantification of G-overhang signal in *pol α* (B) and *icu2-1* (C) mutants. The signals were normalized to the respective wild-type controls. Error bars represent SDs from three (wt-C24, *pol α*, wt-En-2) or four (*icu2-1*) independent samples. (D) Detection of blunt-ended telomeres by the hairpin ligation assay [31]. Ligation of a blunt-ended hairpin to chromosome ends covalently links the complementary telomeric DNA strands. Such cross-linked strands were separated from bulk of TRFs by alkaline electrophoresis and detected by Southern hybridization with the (TTTAGGG)_4_ probe. Control reactions without the hairpin and in which the hairpin was cleaved by *Bam*HI are shown. The arrow-heads indicate signal from the blunt-ended telomeres. Asterisks indicates signal from intrachromosomal telomeric DNA. (E) Frequency of telomeric sequence permutations forming the termini of blunt-ended telomeres. Error bars indicate SDs from four biological replicates.

To assess the impact of DNA polymerase α mutations on telomere maintenance, we performed TRF analysis. The telomeres in *pol* α mutants were overall shorter and more heterogeneous than telomeres in the corresponding wild-type plants (C24) (Figure 5A). A very similar TRF profile is characteristic for *stn1* mutants. A slightly distinct telomere length profile was observed in *icu2-1* mutants. While the *icu2-1* telomeres were substantially shorter (∼3 kb) than in wild-type plants (En-2, ∼6 kb), the heterogeneity was not as pronounced as in *pol α*or *stn1* mutants. Nevertheless, both *icu2-1* and *pol α*alleles have a strong impact on telomere maintenance, although the effect of *icu2-1* seems to be less detrimental. Because DNA polymerase α is required for the fill-in synthesis of the telomeric C-strand, we carried out *in gel* hybridizations to determine the relative size of the telomeric G-overhangs. Interestingly, neither *icu2-1*, nor *pol α* mutants yielded a discernible increase in the G-overhang signal (Figure 5B,C and S5). This suggests that both mutants are largely proficient in C-strand synthesis and that the observed telomere dysfunction is not primarily related to G-overhang maturation.

This result was unexpected considering the strong parallels between *pol α*and *stn1* phenotypes. Therefore, we next examined whether DNA polymerase *α* deficiency affects the structure of blunt-ended telomeres. As anticipated, we readily detected blunt-ended telomeres in *pol α*and *icu2-1* mutants by the hairpin ligation assay, in which the complementary strands of the blunt-ended telomere are joined by a hairpin and then separated by alkaline gel electrophoresis (Figure 5D)}[31]. Sequence analysis of the telomeric blunt-ends by Illumina sequencing revealed that the TTTAGGG-3′ permutation is even more prevalent in C24 than in Col-0 wild-type plants (58% vs. 42%, respectively). Importantly, the *pol α* allele, which is in the C24 genetic background, showed a ∼14% reduction of TTTAGGG-3′ in favor of the GTTTAGG-3′ and GGTTTAG-3′ permutations (Figure 5E; P<0.0001; two-tailed Student's t-test). This is reminiscent of the situation in *stn1* mutants where the abundance of the TTTAGGG-3′ termini was reduced by ∼16% (Figure 2E). The remarkable similarity in telomere-related phenotypes between stn1 and *pol α* mutants supports the notion that STN1 and DNA polymerase αmay act in the same telomere maintenance processes. Interestingly, *stn1 pol α* double mutants exhibit more severe developmental defect and genome instability than each mutation individually, suggesting partially complementary functions of STN1 and DNA polymerase α (Figure S6).

### STN1 is important for the re-replication of non-telomeric loci

**Figure 6:**
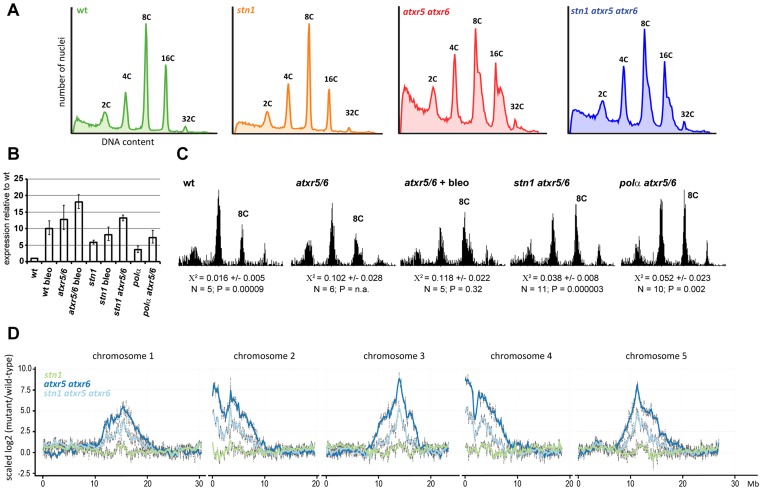
STN1 facilitates re-replication of heterochromatic regions. (A) Representative flow cytograms of the fluorescence intensity of nuclei extracted from the 5^th^ and 6^th^ true leaves of 4 weeks old plants. At least 10.000 nuclei were analyzed per sample. (B) qRT-PCR analysis of *PARP1* expression in two-week old seedlings grown on agar plates with or without Bleomycin (50 ng/mL). Error bars represent SDs from three biological replicates. (C) Representative flow cytograms of nuclei isolated from two week old seedlings; 3000 nuclei from several pooled seedlings were analyzed in each sample. Average X^2^ and its SD obtained by testing the normality of the distribution of 8C nuclei in N samples is shown. X^2^<0.04 indicates that the peak has a normal distribution at the 95% confidence level. P values indicate significance of the difference of the X^2^ statistic from *atrx5 atrx6*. (D) The scaled log2 ratios of genomic DNA Illumina reads from mutant 8C to wild type 8C nuclei are plotted across the chromosomes. The *atrx5 atrx6* and *stn1 atrx5 atrx6* data are derived from sequencing two independent samples.

Analysis of *Arabidopsis* DNA polymerase α mutants showed that an impaired function of a general replication factor may result in very specific telomere-related phenotypes. This prompted us to ask whether STN1 function is limited only to telomeres or whether it plays a broader role in DNA replication. To address this question, we took advantage of *Arabidopsis* mutants lacking the histone methyltransferases ATXR5 and ATXR6 that are responsible for the histone H3K27 monomethylation [40]. The *atxr5 atxr6* double mutants exhibit a higher content of nuclear DNA due to re-replication of heterochromatic regions, which is particularly apparent in cells undergoing endoreplication [41]. We hypothesized that if STN1 plays a role in genome-wide replication, its absence could reduce the re-replication. We generated *stn1 atxr5 atxr6* plants and measured the impact of STN1 deficiency on the nuclear DNA content by flow cytometry. In *Arabidopsis*, leaf development is accompanied by massive endoreplication giving rise to polyploid nuclei with DNA content ranging from 2C to 32C (Figure 6A). A fraction of the endoreplicated nuclei in *atxr5 atxr6* plants have a higher DNA content than nuclei of wild-type leaves, which is detectable on the flow cytometry profiles as broader peaks with a shoulder towards higher DNA content (Figure 6A). Although the shoulder was also apparent in the *stn1 atxr5 atxr6* mutants, it was less pronounced than in *atxr5 atxr6* plants.

STN1 deficiency causes telomere dysfunction resulting in permanent genotoxic stress. Therefore, we examined whether the reduced re-replication in *stn1 atxr5 atxr6* mutants is specifically associated with STN1 dysfunction or whether is it a consequence of DNA damage checkpoint activation. To induce chronic genotoxic stress, we grew *Arabidopsis* seedlings for 14 days on agar plates supplemented with a moderate dose of bleomycin, a radiomimetic drug that induces DNA breaks. Elevated levels of *PARP1* confirmed that this treatment induces constitutive genotoxic stress in wild type seedlings (Figure 6B). Interestingly, *PARP1* expression was highly elevated in non-treated *atxr5 atxr6* seedlings and the expression was further increased by bleomycin. These data indicate that loss of *atxr5* and *atxr6* already results in a permanent DNA damage stress. To quantitatively measure DNA re-replication, we tested how the shape of 8C peaks detected by flow cytometry deviates from the Gaussian distribution. While 8C peaks in wild type seedlings perfectly matched a Gaussian distribution, 8C peaks in *atxr5 atxr6* plants strongly deviate from the normal distribution due to the shoulder containing nuclei with higher DNA content (Figure 6C). Quantitative analysis of 8C peaks in bleomycin treated *atxr5 atxr6* seedlings did not show any reduction of re-replication, indicating that further genotoxic stress does not limit re-replication. In contrast, 8C nuclei in *stn1 atxr5 atxr6* seedlings exhibited an almost normal distribution as the average Χ^2^ for peak normality was significantly reduced in comparison to *atxr5 atxr6* mutants, albeit it was still higher than in wild-type and *stn1* plants (Figure 6C). These data argue that reduced re-replication by STN1 inactivation is not a result of a general DNA checkpoint activation. In addition, we found that *pol α*mutation also impedes re-replication in *atxr5 atxr6* plants, further corroborating the multiple phenotypic parallels between *stn1* and *pol α*.

To determine whether STN1 is required for re-replication of particular genomic regions, we used Illumina sequencing to analyze genomic DNA from sorted 8C nuclei and quantified the density of reads mapped along the individual *Arabidopsis* chromosomes. We confirmed the previously published data on enrichment of pericentric DNA in the nuclei from *atxr5 atxr6* plants over wild-type (Figure 6B). The re-replication of heterochromatic DNA occurred to a much lesser extent in the *stn1 atxr5 atxr6* mutants. More precisely, we observed an overall decline in the enrichment of reads across the re-replicated regions and did not detect any obvious effect on specific chromosomal loci.

## Discussion

CST was identified in *S. cerevisiae* where it protects the 5′ chromosome termini from resection. Consequently, inactivation of any CST subunit is lethal due to the excessive degradation of telomeric and subtelomeric regions along with the activation of a strong DDR [11], [42], [43]. These observations led to the conclusion that CST is an essential component of the telomere protective cap in *S. cerevisiae*. This seems to be the case also in fission yeast where Stn1 and Ten1 are crucial for cell survival and their deletion leads to the loss of telomeric DNA and chromosome end-to-end fusions [44]. The recent identification of the CST complex in higher eukaryotes raised the question of how conserved the telomere-capping function of CST is. While human CST knock-down cell lines produced a range of telomere-associated phenotypes, this was not accompanied by widespread telomere deprotection as judged from the frequency of telomere dysfunction-induced foci and the mild effects on cell viability and proliferation [18], [22], [24]. In addition, the observed ATM/ATR-dependent DDR was not immediately activated after CST impairment but appeared gradually after several population doublings [21]. This argues against a direct role of mammalian CST in telomere capping.

The structural features of telomeric DNA pose a substantial challenge to the progression of the replication machinery, rendering this region vulnerable to fork stalling [45], [46]. CST-depleted human chromosomes were reported to accumulate multi-telomeric signals that are hallmarks of fragile sites within telomeres and which form under conditions of replication stress [20], [21]. In addition, the kinetics of BrdU incorporation demonstrated that CTC1/STN1 depletion slows the replication through telomeric tracks [19], [20], [21]. Thus, it has been proposed that, in contrast to yeast, the mammalian CST complex contributes to the maintenance of telomere integrity via promoting replication of the duplex telomeric DNA and not by providing an end-capping function.

Disruption of the CST components in *Arabidopsis* results in more pronounced telomere-related phenotypes than downregulation of the complex in human cell lines [15], [26], [27]. Some of the phenotypes, such as elevated telomeric recombination, increase in G-overhang size and appearance of chromosome end-to-end fusions, are considered to be hallmarks of telomere deprotection. This led to the suggestion that, in plants, CST contributes to chromosome end-capping. Consistent with this idea, we demonstrate that the growth defects and genome instability are aggravated by EXO1, a nuclease responsible for resecting exposed chromosome termini in a variety of settings and organisms including *Arabidopsis* [11], [31], [47]. This is similar to the situation in budding yeast where mutations reducing DSB resection rescue the lethality arising from Cdc13 inactivation [11], [42], [48] and further substantiates parallels between CST functions in yeast and plants.

Nevertheless, three lines of observations argue against the interpretation that STN1 mediates merely telomere capping in plants. First, we show that telomeric defects in *Arabidopsis stn1* mutants are mitigated by long telomeres. G1 *stn1_(W/C)_* plants that have longer telomeres exhibit only mild telomere dysfunction compared to G1 *stn1_(C)_* plants that have shorter telomeres. Second, although EXO1 exacerbates telomere dysfunction upon STN1 depletion, it does not cause apparent DNA degradation at the bulk of telomeres. Finally, STN1 inactivation leads to aggravated telomere shortening, indicating that chromosome end deprotection is primarily caused by insufficient maintenance of telomeric DNA. Mechanistically, loss of telomeric DNA may be promoted by de-repressing homologous recombination leading to telomere rapid deletion [49]. Indeed, the higher level of t-circles detected in *Arabidopsis* CST mutants supports this scenario [15], [26], [27]. However, comparable levels of t-circle excision have also been detected in *Arabidopsis ku* mutants where it does not result in telomere loss and deprotection [39]. Alternatively, as in mammals, *Arabidopsis* CST may facilitate replication through duplex telomeric DNA and the telomere shortening associated with CST dysfunction may be due to frequent collapse of, or failure to restart, stalled replication forks. This notion is supported by a similar set of phenotypes observed in *Arabidopsis stn1* and *pol α* mutants with respect to telomere structure and protection. Furthermore, DNA damage signaling in *Arabidopsis* CST mutants largely depends on ATR, but not on ATM, an observation consistent with a role for CST in replication [28], [29].

A surprising result of this study is that EXO1 accelerates telomere dysfunction in *stn1* mutants without causing apparent resection at the majority of telomeres. Although we detected signatures of EXO1 activity in the form of altered frequencies of sequence permutations at chromosome termini, EXO1 does not seem to contribute to the G-overhang extension in STN1-null plants. We propose that while EXO1 may gain access to STN1-depleted telomeres, for example at the sites of collapsed replication forks, its activity may be greatly limited in the context of long telomeres by telomere binding proteins [50]. EXO1 activity may be unleashed at short telomeres that lack sufficient amounts of telomere binding proteins, causing excessive degradation of the remaining telomeric and subtelomeric DNA, and triggering a strong DNA damage response and chromosome end-to-end fusions. Consistent with this idea, inactivation of EXO1 in *Arabidopsis* limits DNA damage signaling as well as the frequency of telomere fusions in the context of short telomeres.

The molecular mechanism by which CST facilitates telomere maintenance is still unknown. CST associates with DNA polymerase α and stimulates its processivity and affinity to ssDNA *in vitro* [34], [51]. It has been proposed that CST might recruit DNA polymerase α to help restart collapsed replication forks and to promote the C-strand fill-in synthesis during G-overhang maturation [13], [14], [20], [21]. Our data showing shorter G-overhangs in *Arabidopsis stn1 tert* and *exo1 stn1 tert* mutants indicate that the long G-overhangs in STN1-deficient plants are partially derived from an impaired C-strand fill-in or aberrant processing after telomerase elongation. Strikingly, the hypomorphic DNA polymerase α mutations described in this study appear to be proficient in the C-strand fill-in reaction, while still causing aberrant telomere length maintenance. Considering the essential role of polymerase α in DNA replication, *pol α* and *icu2-1* alleles are expected to produce largely functional proteins capable of initiating Okazaki fragment replication. Thus, *pol α* and *icu2-1* may represent separation-of-function mutations that affect only dispensable activities of polymerase α, such as the restart of collapsed replication forks [52], which may be relevant to the telomere maintenance defects in these mutants. This functional separation draws parallels with the recent observation that CST promotes telomere replication and G-overhang maturation through distinct mechanisms [23].

Our discovery that hypomorphic alleles of DNA polymerase α partially recapitulate the telomere-phenotypes of STN1-deficient plants shows that the malfunction of a general DNA replication factor may lead to very specific defects at telomeres. This raises the question of whether the function of the CST complex is specifically tailored to telomeres, or whether it has a more general role. Although CST does not localize to replication loci [16], it was shown to contribute to genome-wide replication restart after hydroxyurea treatment in human cells [20]. This observation led to the suggestion that CST may be a specialized replication factor that is needed under conditions of replication stress. Non-telomeric function of STN1 is further inferred from a study in budding yeast, where overproduction of Stn1 led to its localization to nontelomeric loci and sensitized the cells to replicative stress in polymerase αdependent manner [53]. In this study, we took advantage of unscheduled re-replication of heterochromatic regions in *Arabidopsis atxr5 atxr6* mutants [41] to functionally assay the role of STN1 in DNA replication. Elevated PARP1 level in *atxr5 atxr6* plants indicates that these mutants experience chronic DNA damage stress, which can be further increased by bleomycin treatment. Thus, our finding that STN1 promotes re-replication in these settings supports data in human cells suggesting a function for the CST complex in facilitating DNA synthesis under replication stress [20]. A further inference is that CST function in genome maintenance is not limited to telomeres; they may just represent a genomic region where the consequence of CST malfunction is phenotypically most apparent.

## Material and Methods

### Plant material

The following *Arabidopsis thaliana* mutant lines were used in this study: *stn1-1* [26], *tert-1* [33], *exo1a-1* and *exo1b-1* [31], *pol α* [37], *icu2-1* [36] and *atxr5 atxr6* [40]. The primers used for PCR genotyping are listed in Table S1. The plants were grown at 22°C with 16/8 h light/dark period.

### Assays for determining the telomere structure

The DNA was extracted from four to six weeks old plants as previously described [31]. Terminal restriction fragment (TRF) analysis with *Tru9*I restriction enzyme [54], t-circle amplification assay [39], hairpin-ligation assay [31] and fusion PCR [38] were performed according to published protocols. Telomere length was determined from TRF blots by using TeloTool, software for quantitative analysis of TRF data [55]. Mitotic spreads for quantification of anaphase bridges were prepared from *Arabidopsis* floral buds as previously described [32]. The frequency of anaphase bridges was calculated by scoring at least 200 anaphases per plant; at least three plants were analyzed for each category to calculate average frequency and SD.

### G-overhang analysis

The *in gel* hybridization was performed as earlier described [56]. We used *Hind*III to generate long TRFs in order to minimize effect of telomere length and heterogeneity on signal detection. For the control reactions, the DNA was pre-treated with 30 units T4 DNA polymerase (New England Biolabs) for 30 min at 37°C to remove the 3′ G-overhangs. The hybridization signals were scanned using the Molecular Imager PharosFX Plus (BioRad) and quantified with the Image Lab software (BioRad). To correct for loading differences, the hybridization signals were normalized to the ethidium bromid staining of the agarose gels. The G-overhang signal was obtained after subtracting the signal from the T4 DNA Pol pretreated samples. Finally, the G-overhang signal of the wild type samples was set to one and all other samples were normalized to this value. We usually perform quantitative comparisons of samples run in the same gels to minimize experimental variation.

### Terminal sequence permutation analysis

The analysis was performed as previously described with small modifications [31]. Genomic DNA (1 µg) was ligated with the hairpin blunt HP 3 (Table S1) at 16°C, followed by heat inactivation. The samples were digested with 20 U *Tru*1I (Fermentas) overnight at 65°C, ethanol precipitated and dissolved in 25 µl TE. 5 µl of the reaction were used as a template for 20 cycles of PCR amplification in 1xGo*Taq* buffer (Promega) supplemented with 0.2 mM dNTPs, 0.5 µM of (TTTAGGG)_4_ primer,0.5 µM HP 3 primer and 2.5 U Go*Taq* (Promega) in a volume of 50 µl. The PCR products were ethanol precipitated, digested with *Alu*I and purified with the Nucleospin column purification kit (Macherey-Nagel). The library for Illumina sequencing was prepared with the NEB Next DNA library Prep Reagent Set (New England Biolabs) according to the manufacturer's instruction using 100 ng of digested DNA. The samples were sequenced with single-end 50 bp reads using the Illumina HiSeq 2000. Per sequencing lane, eight independent samples were pooled each carrying a unique barcode (Table S1). Per barcode, 1.1×10^6^–3.1×10^6^ reads were obtained and analyzed with the custom-made program “TELOMERATOR” [31].

### Flow cytometry and Illumina sequencing of sorted nuclei

The nuclei were prepared from 1.5 g mature rosette leaves (3^rd^–8^th^) collected from 3–4 week old plants. The leaves were chopped on ice in 5 ml isolation buffer (15 mM Tris-HCl pH = 7.5, 2 mM EDTA, 0.5 mM Spermidine, 20 mM NaCl, 80 mM KCl, 15 mM β-mercaptoethanol, 0.1% Triton X-100, pH 7.5). The released nuclei were purified by filtration through a 50 µm CellTrics disposable filter (Partec) and collected by centrifugation at 2800×g at 4°C. The nuclei were resuspended in 600 µl isolation buffer, filtered through a clean 50 µm filter and stained with 400 µl CyStain UV buffer (Partec). After a final filtration through a 40 µm BD Falcon cell strainer (BD Biosciences), the stained nuclei were sorted with the BD FACS Aria III (BD biosciences) using an 85 µM nozzle with sheath pressure at 45 psi and a threshold rate of ∼3000 events/s. The DNA of the 8C fraction was extracted by incubating 500.000 sorted nuclei in 200 µl lysis buffer (150 mM NaCl, 10 mM Tris-HCl pH = 8.0, 100 mM EDTA, 1% SDS) for 10 min at 65°C while shaking. The RNA was removed by RNase A treatment followed by incubation with Proteinase K (Thermo Scientific). The DNA was purified by phenol:chlorophorm:isoamyl alcohol (25∶24∶1, v/v) extraction as well as ethanol precipitation. Finally, the DNA was sonicated to an average fragment size of ∼200 bp using the Covaris S2.20 ng DNA were used to prepare the library for Illumina sequencing with the NEXTflex ChIP-Seq Kit (Bioo Scientific). NEXTflex-96 DNA barcodes (Bioo Scientific) were used to sequence four independent samples per lane with single-end 100 bp reads using the Illumina HiSeq2000. For flow cytometry, nuclei from several 2 week old seedlings were prepared according to a scaled-down version of the protocol described above. The fluorescence intensity of nuclei was measured with a CyFlow counter (Partec) and their distribution as well as statistical analysis of 8C peaks was done using FlowMax software (Partec).

### Analysis of DNA re-replication

The sequencing reads were aligned to the *A. thaliana* genome (TIAR 10) using Bowtie2 [57]. For reads mapping to multiple positions, the best (or randomly selected in case of ties) alignment was used. Multiple reads mapping to the same position were collapsed to one read. The method used for analyzing re-replicated regions derived from the strategy described by Stroud et al. [58]. The genome was tiled into 10 kb bins and the reads in the bins were summed up. Bins with a mappability of 0 and bins with more reads than the top 1% of all bins plus the median were removed. The mappability was calculated using the GEM toolbox for 100 bp reads [59]. For the *atxr5 atxr6* and *atxr5 atxr6 stn1* mutants where two independent samples were sequenced, the bins were averaged between replicates. For all bins, the log2 ratio to the wild type was calculated and then scaled to account for differences in read numbers and base replication ratio by finding the minimum mean ratio in a 5 Mb sliding window for each chromosome. The mean of the base window was subtracted from each bin and the result divided by the standard deviation to obtain a scaled value that is 0 for a base replication rate and varies according to the variability of each sample (higher overall variability is penalized). In the figure, all bins were additionally smoothed by calculating a running mean over 10 bins. The smoothing lines were produced by cubic regression splines.

## Supporting Information

Figure S1 Progressive onset of telomere dysfunction in *stn1* mutants. (A) Frequency of phenotypic categories as defined in Fig. 1A in consecutive generations of *stn1* mutants from a mixed Col-0/WS background [31]. The number of plants scored is indicated in parentheses. The majority of plants in G1 exhibit no or only mild growth defects, but the frequency of plants with severe growth defects increases in subsequent generation. Telomere length in G1 *stn1_(w/c)_* plants averages 2.78 kb (SD = 0.20; N = 2) while only 1.98 kb (SD = 0.47; N = 2) in G2 *stn1_(w/c)_*. Telomere length data were obtained by quantification of the TRFs blots in Fig. S7B published in [31]. (B) Representative examples of *stn1* mutants in a pure Col-0 background. (C) Frequency of phenotypic categories in *stn1* mutant populations in a pure Col-0 background. Number of plants scored is indicated in parentheses; only three viable plants were recovered in G3. Average telomere length in G1 *stn1_(C)_* plants determined from the TRF blot in the Figure 2A is 2.20 kb (SD = 0.78; N = 2), which is shorter than in G1 *stn1_(w/c)_* plants. (D,E) Amount of telomeric DNA in *stn1_(C)_* plants measured by dot-blot hybridization. (D) Dot blot autoradiograms with telomeric and CEN180-repeat probes. (E) Quantification of dot blots in (D). DNA loading was assessed from the CEN180 signal. Error bars represent SDs from two (G1 and G3) or three independent DNA samples (G2).
(JPG)

Figure S2 EXO1 is not required for t-circle excision in *stn1* mutants. (A) T-circle amplification assay. Signals from t-circles (arrow), TRFs and interstitial telomeric DNA (asterisks) are indicated. (B) Relative quantification of the t-circles signal from (A).
(JPG)

Figure S3 Analysis of telomerase-deficient *stn1* mutants. Crossing strategy used to generate (A) *tert stn1* and (B) *exo1 tert stn1* mutants. Wild type and mutant alleles are indicated by capital and small letters, respectively (A – *EXO1A*, B – *EXO1B*, S – *STN1, T - TERT*). (C) Representative G1 *stn1 tert* and G1 *exo1 stn1 tert* plants. (D) G-overhang analysis by the *in gel* hybridization technique. DNA samples pretreated with T4 DNA polymerase to remove 3′ G-overhangs are indicated (3′ exo). The gels were first hybridized under nondenaturing conditions (top panels) and then denatured and hybridized again (bottom panels).
(JPG)

Figure S4 DNA polymerase α mutants. (A) Schematic representation of the *A. thaliana* catalytic subunit of DNA polymerase α with indicated amino acid substitutions in *pol α* and *icu2-1* alleles. (B) Approximately five weeks old DNA polymerase α mutant plants with corresponding wild-types. Detailed pictures of inflorescences with siliques (seed pods) are shown in the bottom panel. Note that *pol α* mutants are smaller than *icu2-1* mutants and exhibit stem fasciation like *stn1* plants.
(JPG)

Figure S5 G-overhang analysis of *pol α* and *icu2-1* mutants by the *in gel* hybridization technique. DNA samples pretreated with T4 DNA polymerase to remove 3′ G-overhangs are indicated (3′ exo). The gels were first hybridized under nondenaturing conditions and then denatured and hybridized again.
(JPG)

Figure S6 Analysis of *stn1 pol α*double mutants. (A) Picture of approximately six weeks old mutants. Detailed picture of *stn1 pol α* double mutants is shown in the inset. Stn1 mutants show only relatively mild phenotypes, which may be due to longer telomeres in the Col-0/C24 mixed background. While plants homozygous for the *pol α* allele are fertile and produce only a few small leaves partially due to early flowering, *stn1 pol α* mutants are similar to terminal *stn1* plants and do not produce viable seeds. (B) Frequency of anaphase bridges in cells from floral tissues of the first generation mutant plants. Error bars represent SDs from three independent samples. 200-400 anaphases were scored per sample.
(JPG)

Table S1 List of oligonucleotides.
(DOCX)
