# Supplementary figures and images for: Role of STN1 and DNA Polymerase α in Telomere Stability and Genome-Wide Replication in Arabidopsis
Source: PLoS Genet. 2014 Oct 9;10(10):e1004682. doi: 10.1371/journal.pgen.1004682 (PMC4191939; doi:10.1371/journal.pgen.1004682)

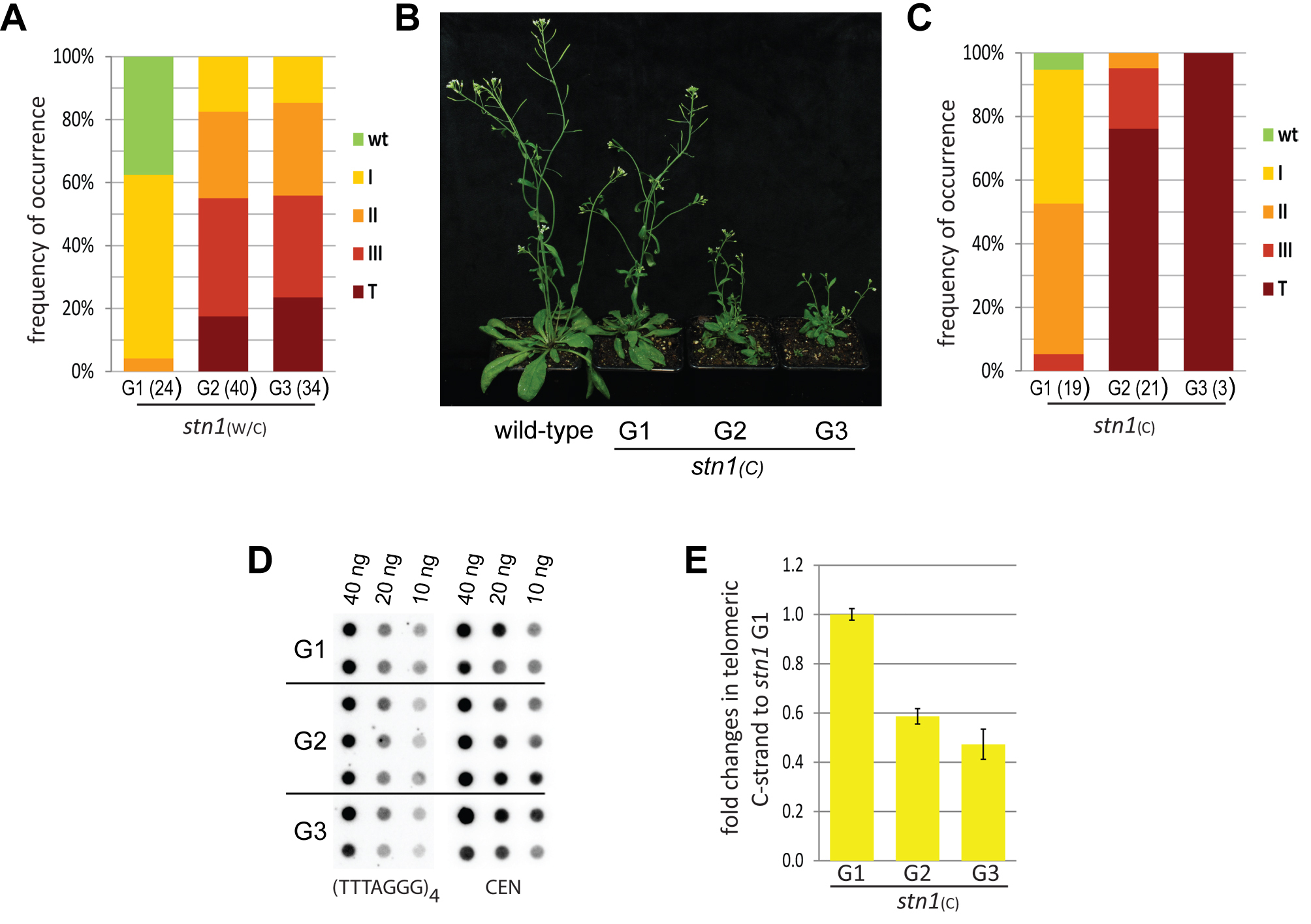

Supplement: Figure S1 — Progressive onset of telomere dysfunction in stn1 mutants. (A) Frequency of phenotypic categories as defined in Fig. 1A in consecutive generations of stn1 mutants from a mixed Col-0/WS background [31]. The number of plants scored is indicated in parentheses. The majority of plants in G1 exhibit no or only mild growth defects, but the frequency of plants with severe growth defects increases in subsequent generation. Telomere length in G1 stn1(w/c) plants averages 2.78 kb (SD = 0.20; N = 2) while only 1.98 kb (SD = 0.47; N = 2) in G2 stn1(w/c). Telomere length data were obtained by quantification of the TRFs blots in Fig. S7B published in [31]. (B) Representative examples of stn1 mutants in a pure Col-0 background. (C) Frequency of phenotypic categories in stn1 mutant populations in a pure Col-0 background. Number of plants scored is indicated in parentheses; only three viable plants were recovered in G3. Average telomere length in G1 stn1(C) plants determined from the TRF blot in the Figure 2A is 2.20 kb (SD = 0.78; N = 2), which is shorter than in G1 stn1(w/c) plants. (D,E) Amount of telomeric DNA in stn1(C) plants measured by dot-blot hybridization. (D) Dot blot autoradiograms with telomeric and CEN180-repeat probes. (E) Quantification of dot blots in (D). DNA loading was assessed from the CEN180 signal. Error bars represent SDs from two (G1 and G3) or three independent DNA samples (G2). (JPG) [file pgen.1004682.s001.jpg]

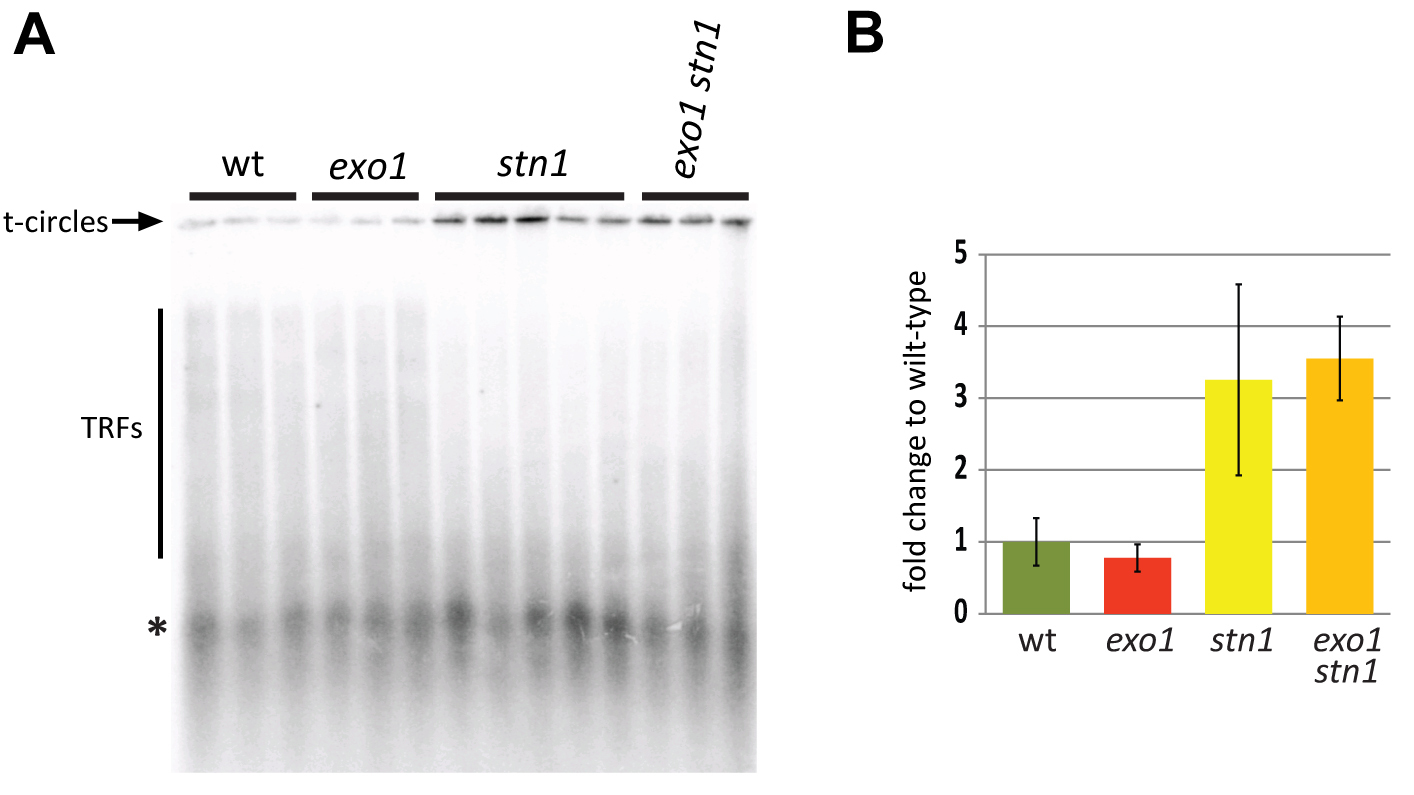

Supplement: Figure S2 — EXO1 is not required for t-circle excision in stn1 mutants. (A) T-circle amplification assay. Signals from t-circles (arrow), TRFs and interstitial telomeric DNA (asterisks) are indicated. (B) Relative quantification of the t-circles signal from (A). (JPG) [file pgen.1004682.s002.jpg]

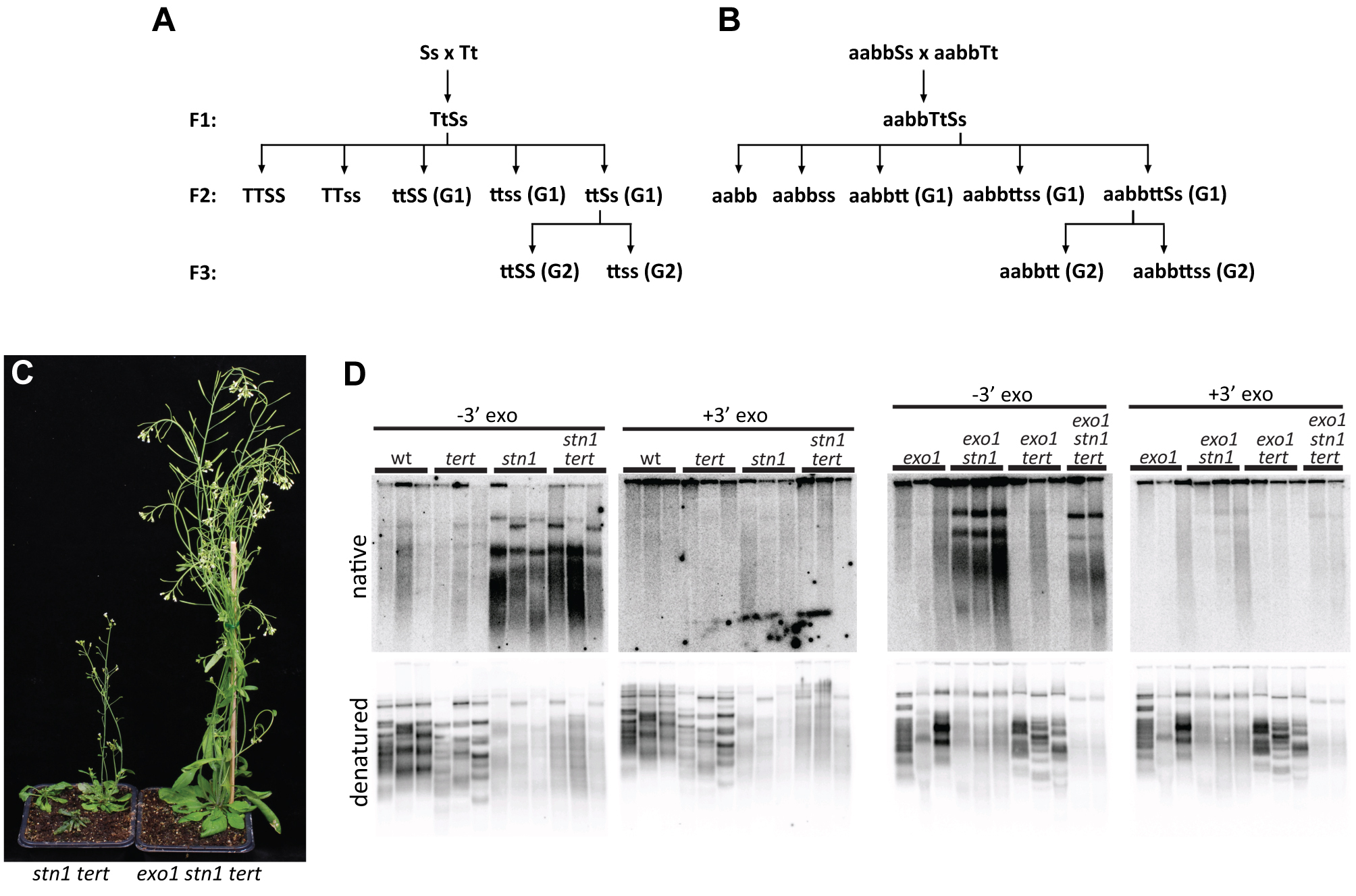

Supplement: Figure S3 — Analysis of telomerase-deficient stn1 mutants. Crossing strategy used to generate (A) tert stn1 and (B) exo1 tert stn1 mutants. Wild type and mutant alleles are indicated by capital and small letters, respectively (A – EXO1A, B – EXO1B, S – STN1, T - TERT). (C) Representative G1 stn1 tert and G1 exo1 stn1 tert plants. (D) G-overhang analysis by the in gel hybridization technique. DNA samples pretreated with T4 DNA polymerase to remove 3′ G-overhangs are indicated (3′ exo). The gels were first hybridized under nondenaturing conditions (top panels) and then denatured and hybridized again (bottom panels). (JPG) [file pgen.1004682.s003.jpg]

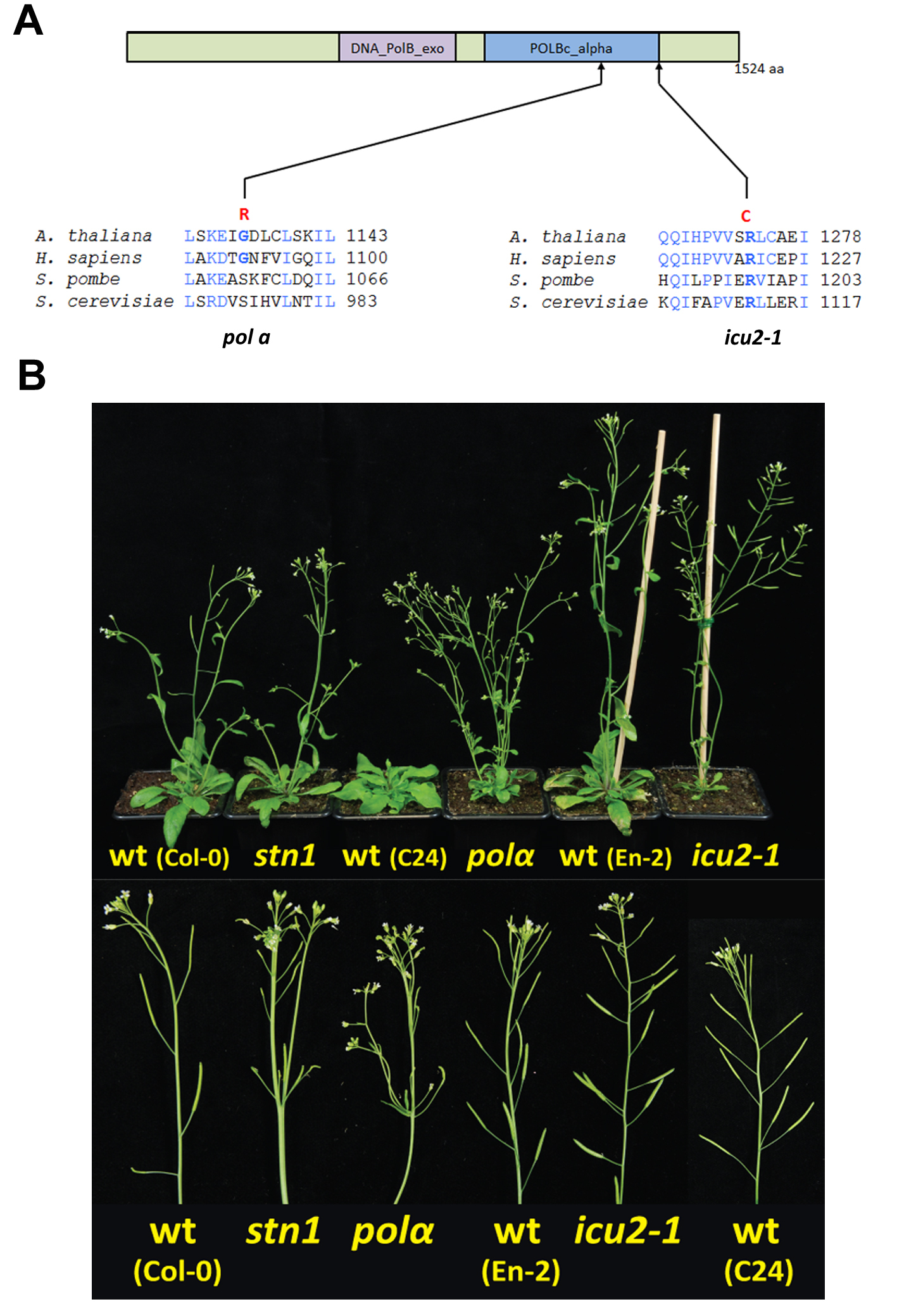

Supplement: Figure S4 — DNA polymerase α mutants. (A) Schematic representation of the A. thaliana catalytic subunit of DNA polymerase α with indicated amino acid substitutions in pol α and icu2-1 alleles. (B) Approximately five weeks old DNA polymerase α mutant plants with corresponding wild-types. Detailed pictures of inflorescences with siliques (seed pods) are shown in the bottom panel. Note that pol α mutants are smaller than icu2-1 mutants and exhibit stem fasciation like stn1 plants. (JPG) [file pgen.1004682.s004.jpg]

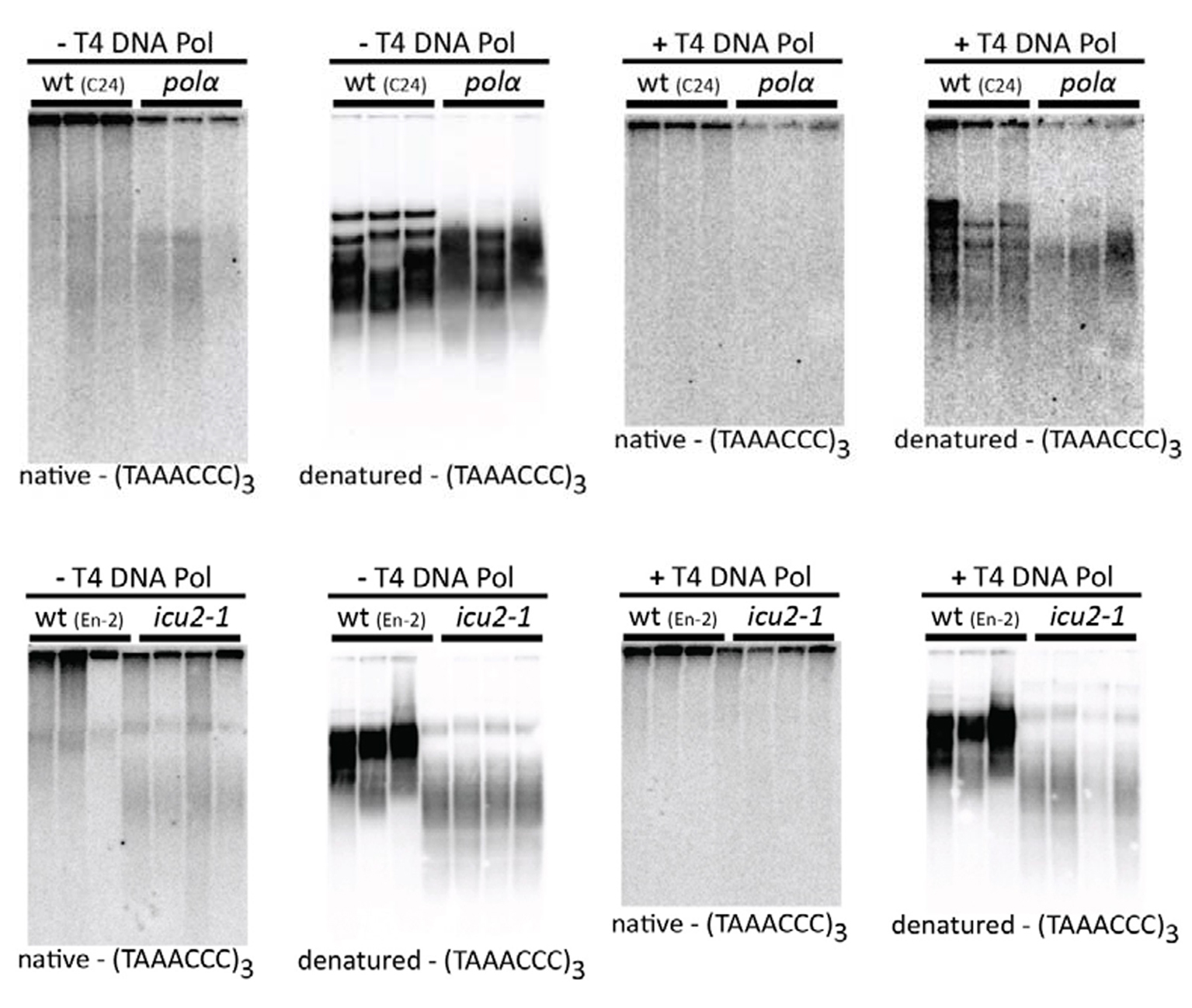

Supplement: Figure S5 — G-overhang analysis of pol α and icu2-1 mutants by the in gel hybridization technique. DNA samples pretreated with T4 DNA polymerase to remove 3′ G-overhangs are indicated (3′ exo). The gels were first hybridized under nondenaturing conditions and then denatured and hybridized again. (JPG) [file pgen.1004682.s005.jpg]

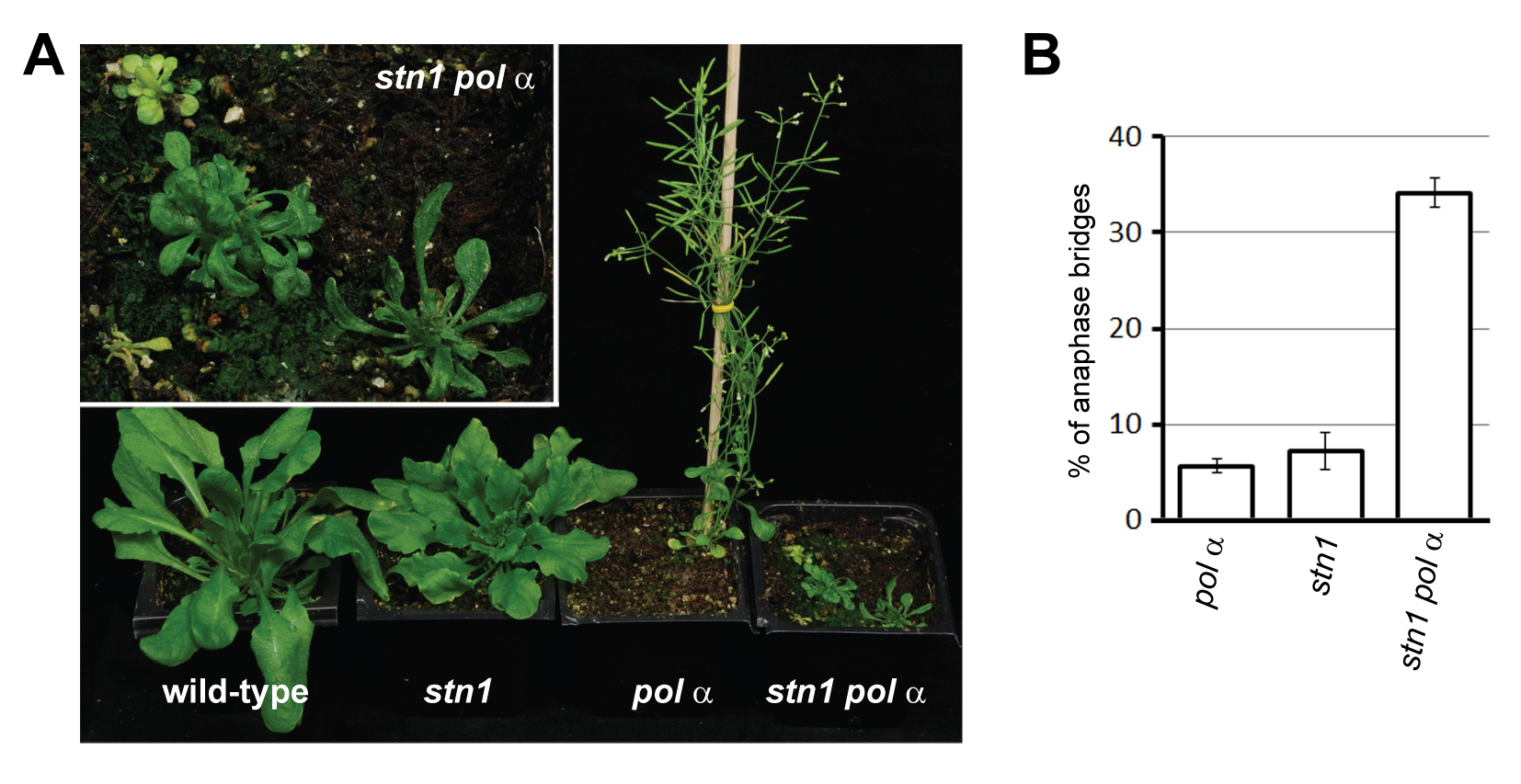

Supplement: Figure S6 — Analysis of stn1 pol αdouble mutants. (A) Picture of approximately six weeks old mutants. Detailed picture of stn1 pol α double mutants is shown in the inset. Stn1 mutants show only relatively mild phenotypes, which may be due to longer telomeres in the Col-0/C24 mixed background. While plants homozygous for the pol α allele are fertile and produce only a few small leaves partially due to early flowering, stn1 pol α mutants are similar to terminal stn1 plants and do not produce viable seeds. (B) Frequency of anaphase bridges in cells from floral tissues of the first generation mutant plants. Error bars represent SDs from three independent samples. 200-400 anaphases were scored per sample. (JPG) [file pgen.1004682.s006.jpg]
