# Supplementary material for: Role of STN1 and DNA Polymerase α in Telomere Stability and Genome-Wide Replication in Arabidopsis
Source: PLoS Genet. 2014 Oct 9;10(10):e1004682. doi: 10.1371/journal.pgen.1004682 (PMC4191939; doi:10.1371/journal.pgen.1004682)
Supplement: Table S1 — List of oligonucleotides. (DOCX) [file pgen.1004682.s007.docx]

**Supplementary Table S1.** List of oligonucleotides

| **Primer** | **Sequence [5’-3’]** | **Application** |
| --- | --- | --- |
| Stn1_s | ACGCCTGACACAGTCACCTAC | Genotyping *stn1-1* |
| Stn1_r2 | GCATCACTCCTCTGTACGAGC | Genotyping *stn1-1* |
| Lba-1 | ATGGTTCACGTAGTGGGCCTCG | Genotyping *stn1-1* |
| Tert30 | cacaccattggcctttgg | Genotyping *tert-1* |
| Tert45 | CCATTCATTGCTCAAGTCTC | Genotyping *tert-1* |
| LB-CD6 | GAACATCGGTCTCAATGCAA | Genotyping *tert-1* |
| ExoA-1 | CATTCCCGTCCTTCAGATTCGTA | Genotyping *exo1a-2* |
| ExoA-2 | GGACCTCCATCAAAGACCATGAT | Genotyping *exo1a-2* |
| GABI-1 | GATGTTAGGCCAGGACTTTGAA | Genotyping *exo1a-2* |
| ExoB-1 | GCTCATGCATTCATCTCCAAGTA | Genotyping *exo1b-1* |
| ExoB-2 | CCTTCAGCAATTGCAACAGCAA | Genotyping *exo1b-1* |
| Lbd-1 | GAACCACCATCAAACAGGATTT | Genotyping *exo1b-1* |
| Ku80-21 | TCTTCCAGCACAACTCCTCA | Genotyping *ku80-1* |
| Ku80-33 | caagccgataatactccaag | Genotyping *ku80-1* |
| Lbcd-7 | CATTTTATAATAACGCTGCGGACATCTAC | Genotyping *ku80-1* |
| Polα_s | GCAGTTTAAGGATGGGAA | Genotyping *polα* |
| Polα_r | CTTCAACAACATCCTCAC | Genotyping *polα* |
| Icu2-1_s | TCTGTGCAGGGTAATGCT | Genotyping *icu2-1* |
| Icu2-1_r | AAGAGGGAGGTAGAGGGA | Genotyping *icu2-1* |
| Atxr5_s | TGAAATGCCTTAGACCCATTG | Genotyping *atxr5* |
| Atxr5_r | CATGATGCTGTCACAATCGTC | Genotyping *atxr5* |
| LBb1.3 | ATTTTGCCGATTTCGGAAC | Genotyping *atxr5* |
| Atxr6_s | TTGAGATGAATCTGGAGACCG | Genotyping *atxr6* |
| Atxr6_r | GTGAGTGAACTCGGACGAGTC | Genotyping *atxr6* |
| SAIL-LB3 | TAGCATCTGAATTTCATAACCAATCTCGATACAC | Genotyping *atxr6* |
| ATM_f | TGCAGCTGCGTCTCTGCATGA | qRT-PCR of *ATM* |
| ATM_r | CTTCATGCCGCCCTTGGGCA | qRT-PCR of *ATM* |
| ATR_f | AACCCACATGCTCAGCGGGC | qRT-PCR of *ATR* |
| ATR_r | TCAACCGGCGAGCCTGACCT | qRT-PCR of *ATR* |
| BRCA1_ED_f | GTTGCCCTGTGAAGAGGCTAATTC | qRT-PCR of *BRCA1* |
| BRCA1_ED_r | TAGGCTGAGAGTGCAGTGGTTC | qRT-PCR of *BRCA1* |
| PARP1_ED_f | ATGCTACTCTGGCACGGTTCAC | qRT-PCR of *PARP1* |
| PARP1_ED_r | AGGAGGAGCTATTCGCAGACCTTG | qRT-PCR of *PARP1* |
| SAND_qF1 | CAAGGCAGGAAATCACCAGGTTG | qRT-PCR of At2g28390 |
| SAND-qR1 | CTGTACAGCTGATGCAGACCAG | qRT-PCR of At2g28390 |
| (TTTAGGG)_4_ | TTTAGGGTTTAGGGTTTAGGGTTTAGGG | Probe for telomeric C-strand |
| (TAAACCC)_3_ | TAAACCCTAAACCCTAAACCC | Probe for telomeric G-strand |
| CEN-1 | ATCAAGTCATATTCGACTCCA | Forward primer for CEN180-repeat probe |
| CEN-2 | CTCATGTGTATGATTGAGAT | Reverse primer for CEN180-repeat probe |
| 5S_f | ttgggctatattacggaccca | Forward primer for 5S rDNA probe |
| 5S_r | gtcctgcttctttcgtcggag | Reverse primer for 5S rDNA probe |
| 1L_fusion | ACAAGGATAGAAATAGAGCATCGTC | fusion PCR (1L) |
| 1R_1 | CTATTGCCAGAACCTTGATATTCAT | fusion PCR (1R) |
| Pat51-5 | CAACATGGCCCATTTAAGATTGAACG | fusion PCR (2R) |
| 3L_fusion | AGACGAGGAGACTAGGAACG | Fusion PCR (3L) |
| Blunt HP | p-GGATCCGACTTTTGTCGGATCC | Blunt hairpin ligation |
| Blunt HP 3 | p-CCAGCTCTAGATGGTGGTGTTGGTTCTAGAGCTGG | Hairpin used for deep sequencing |
| HP 3 primer | GCTCTAGATGGTGGTGTTGGT | Blunt HP 3 specific primer |
| Illumina Adp1 AGT1 | p-AGTAGATCGGAAGAGCTCGTATGCCGTCTTCTGCTTG | Barcode 1 for analysis of terminal permutation |
| Illumina Adp1 AGT2 | ACACTCTTTCCCTACACGACGCTCTTCCGATCTACTT- Ps | Barcode 1 for analysis of terminal permutation |
| Illumina Adp2 GTC1 | p-GTCAGATCGGAAGAGCTCGTATGCCGTCTTCTGCTTG | Barcode 2 for analysis of terminal permutation |
| Illumina Adp2 GTC2 | ACACTCTTTCCCTACACGACGCTCTTCCGATCTGACT- Ps | Barcode 2 for analysis of terminal permutation |
| Illumina Adp3 TCA1 | p-TCAAGATCGGAAGAGCTCGTATGCCGTCTTCTGCTTG | Barcode 3 for analysis of terminal permutation |
| Illumina Adp3 TCA2 | ACACTCTTTCCCTACACGACGCTCTTCCGATCTTGAT- Ps | Barcode 3 for analysis of terminal permutation |
| Illumina Adp4 CAG1 | p-CAGAGATCGGAAGAGCTCGTATGCCGTCTTCTGCTTG | Barcode 4 for analysis of terminal permutation |
| Illumina Adp4 CAG2 | ACACTCTTTCCCTACACGACGCTCTTCCGATCTCTGT- Ps | Barcode 4 for analysis of terminal permutation |
| Illumina Adp5 TGC1 | p-TGCAGATCGGAAGAGCTCGTATGCCGTCTTCTGCTTG | Barcode 5 for analysis of terminal permutation |
| Illumina Adp5 TGC2 | ACACTCTTTCCCTACACGACGCTCTTCCGATCTGCAT- Ps | Barcode 5 for analysis of terminal permutation |
| Illumina Adp6 CTA1 | p-CTAAGATCGGAAGAGCTCGTATGCCGTCTTCTGCTTG | Barcode 6 for analysis of terminal permutation |
| Illumina Adp6 CTA2 | ACACTCTTTCCCTACACGACGCTCTTCCGATCTTAGT- Ps | Barcode 6 for analysis of terminal permutation |
| Illumina Adp7 ACG1 | p-ACGAGATCGGAAGAGCTCGTATGCCGTCTTCTGCTTG | Barcode 7 for analysis of terminal permutation |
| Illumina Adp7 ACG2 | ACACTCTTTCCCTACACGACGCTCTTCCGATCTCGTT- Ps | Barcode 7 for analysis of terminal permutation |
| Illumina Adp8 GAT1 | p-GATAGATCGGAAGAGCTCGTATGCCGTCTTCTGCTTG | Barcode 8 for analysis of terminal permutation |
| Illumina Adp8 GAT2 | ACACTCTTTCCCTACACGACGCTCTTCCGATCTATCT- Ps | Barcode 8 for analysis of terminal permutation |
| Sequencing primer | ACACTCTTTCCCTACACGACGCTCTTCCGATCT | Universal sequencing primer for analysis of terminal permutation |
| HT Adapter 1 _f | AATGATACGGCGACCACCGAGATCTACACTCTTTCCCTACACGACGCTCTTCCGATCT | NEXTflex-96 Adapter 1 for analysis of re-replication |
| HT Adapter 1 _r | p-GATCGGAAGAGCACACGTCTGAACTCCAGTCACAAC  GTGATATCTCGTATGCCGTCTTCTGCTTG | NEXTflex-96 Adapter 1 for analysis of re-replication |
| HT Adapter 2 _f | AATGATACGGCGACCACCGAGATCTACACTCTTTCCCTACACGACGCTCTTCCGATCT | NEXTflex-96 Adapter 2 for analysis of re-replication |
| HT Adapter 2_r | p-GATCGGAAGAGCACACGTCTGAACTCCAGTCACAAA  CATCGATCTCGTATGCCGTCTTCTGCTTG | NEXTflex-96 Adapter 2 for analysis of re-replication |
| HT Adapter 3_f | AATGATACGGCGACCACCGAGATCTACACTCTTTCCCTACACGACGCTCTTCCGATCT | NEXTflex-96 Adapter 3 for analysis of re-replication |
| HT Adapter 3_r | p-GATCGGAAGAGCACACGTCTGAACTCCAGTCACATG  CCTAAATCTCGTATGCCGTCTTCTGCTTG | NEXTflex-96 Adapter 3 for analysis of re-replication |
| HT Adapter 4_f | AATGATACGGCGACCACCGAGATCTACACTCTTTCCCTACACGACGCTCTTCCGATCT | NEXTflex-96 Adapter 4 for analysis of re-replication |
| HT Adapter 4_r | p-GATCGGAAGAGCACACGTCTGAACTCCAGTCACAGT  GGTCAATCTCGTATGCCGTCTTCTGCTTG | NEXTflex-96 Adapter 4 for analysis of re-replication |
| HT Adapter 5_f | AATGATACGGCGACCACCGAGATCTACACTCTTTCCCTACACGACGCTCTTCCGATCT | NEXTflex-96 Adapter 5 for analysis of re-replication |
| HT Adapter 5_r | p-GATCGGAAGAGCACACGTCTGAACTCCAGTCACACC  ACTGTATCTCGTATGCCGTCTTCTGCTTG | NEXTflex-96 Adapter 5 for analysis of re-replication |
| HT Adapter 6_f | AATGATACGGCGACCACCGAGATCTACACTCTTTCCCTACACGACGCTCTTCCGATCT | NEXTflex-96 Adapter 6 for analysis of re-replication |
| HT Adapter 6_r | p-GATCGGAAGAGCACACGTCTGAACTCCAGTCACACA  TTGGCATCTCGTATGCCGTCTTCTGCTTG | NEXTflex-96 Adapter 6 for analysis of re-replication |
| HT Adapter 7_f | AATGATACGGCGACCACCGAGATCTACACTCTTTCCCTACACGACGCTCTTCCGATCT | NEXTflex-96 Adapter 7 for analysis of re-replication |
| HT Adapter 7_r | p-GATCGGAAGAGCACACGTCTGAACTCCAGTCACCAG  ATCTGATCTCGTATGCCGTCTTCTGCTTG | NEXTflex-96 Adapter 7 for analysis of re-replication |
| HT Adapter 8_f | AATGATACGGCGACCACCGAGATCTACACTCTTTCCCTACACGACGCTCTTCCGATCT | NEXTflex-96 Adapter 8 for analysis of re-replication |
| HT Adapter 8_r | p-GATCGGAAGAGCACACGTCTGAACTCCAGTCACCAT  CAAGTATCTCGTATGCCGTCTTCTGCTTG | NEXTflex-96 Adapter 8 for analysis of re-replication |
